# Climate change amplifies the interactions between wind and bark beetle disturbances in forest landscapes

**DOI:** 10.1007/s10980-016-0396-4

**Published:** 2016-05-23

**Authors:** Rupert Seidl, Werner Rammer

**Affiliations:** Department of Forest- and Soil Sciences, Institute of Silviculture, University of Natural Resources and Life Sciences (BOKU) Vienna, Peter Jordan Straße 82, 1190 Vienna, Austria

**Keywords:** Forest disturbance interactions, Windthrow, *Ips typographus*, Disturbance modeling, Climate change impacts, *Picea abies*

## Abstract

**Context:**

Growing evidence suggests that climate change could substantially alter forest disturbances. Interactions between individual disturbance agents are a major component of disturbance regimes, yet how interactions contribute to their climate sensitivity remains largely unknown.

**Objectives:**

Here, our aim was to assess the climate sensitivity of disturbance interactions, focusing on wind and bark beetle disturbances.

**Methods:**

We developed a process-based model of bark beetle disturbance, integrated into the dynamic forest landscape model iLand (already including a detailed model of wind disturbance). We evaluated the integrated model against observations from three wind events and a subsequent bark beetle outbreak, affecting 530.2 ha (3.8 %) of a mountain forest landscape in Austria between 2007 and 2014. Subsequently, we conducted a factorial experiment determining the effect of changes in climate variables on the area disturbed by wind and bark beetles separately and in combination.

**Results:**

iLand was well able to reproduce observations with regard to area, temporal sequence, and spatial pattern of disturbance. The observed disturbance dynamics was strongly driven by interactions, with 64.3 % of the area disturbed attributed to interaction effects. A +4 °C warming increased the disturbed area by +264.7 % and the area-weighted mean patch size by +1794.3 %. Interactions were found to have a ten times higher sensitivity to temperature changes than main effects, considerably amplifying the climate sensitivity of the disturbance regime.

**Conclusions:**

Disturbance interactions are a key component of the forest disturbance regime. Neglecting interaction effects can lead to a substantial underestimation of the climate change sensitivity of disturbance regimes.

**Electronic supplementary material:**

The online version of this article (doi:10.1007/s10980-016-0396-4) contains supplementary material, which is available to authorized users.

## Introduction

Disturbances are key drivers of landscape dynamics, shaping the structure, composition, and functioning of ecosystems (Turner 2010). Disturbance agents such as wildfire, wind, or insect outbreaks affect landscapes around the globe, disrupting the structure of ecosystems, communities, or populations, and changing their resource availability and physical environment (Pickett and White 1985). Over longer temporal and larger spatial scales individual disturbance events form a disturbance regime, characterized by typical sizes and return intervals of disturbance (Turner et al. 1998). Within disturbance regimes, individual disturbance agents and events are rarely independent of each other but interact in space and time. Interactions exist inter alia between wind and bark beetle outbreaks (Eriksson et al. 2005; Stadelmann et al. 2014), bark beetles and wildfire (Kulakowski et al. 2012; Harvey et al. 2014), as well as drought and bark beetle outbreaks (Netherer et al. 2015; Seidl et al. 2016a). Theory suggests that there are two main pathways of disturbance interactions: Linked interactions, in which a disturbance alters the likelihood, extent, and/or severity of subsequent disturbances; and compound interactions, in which the interaction between disturbances has synergistic effects beyond the sum of the individual disturbances and results in different ecological consequences compared to individual disturbance events (Simard et al. 2011; Buma 2015).

Changes in climate have the potential to strongly alter disturbance regimes. In many ecosystems, the climate regime expected for the future is conducive to an increase in disturbance activity (Westerling et al. 2011; Seidl et al. 2014b). Consequently, an increase in linked disturbance interactions can be expected in these areas. Furthermore, cascading effects of climate change within the disturbance regime are possible, i.e., systems in which changes in climatic drivers influence disturbance agents that are not directly sensitive to these drivers (Buma 2015). For instance, the direct effects of climate change on landslide activity remain poorly understood. Yet, landslides might become more frequent under climate change even in the absence of direct climate effects, as insect disturbances—which are likely to intensify as a result of climate change—have been found to increase landslide risk (Simard and Lajeunesse 2015). A changing climate could also alter synergistic processes between disturbances and modulate compound interactions, and ultimately lead to ecological surprises (Paine et al. 1998). Such changes have the potential to challenge the resilience of forest ecosystems to changing disturbance regimes (Buma and Wessman 2011; Seidl et al. 2016b).

Despite a growing understanding of interacting disturbance agents (Bebi et al. 2003; Eriksson et al. 2005; Kulakowski et al. 2012; Harvey et al. 2014; Stadelmann et al. 2014; Hart et al. 2015) quantifying the climate sensitivity of disturbance interactions remains challenging, not least because experimentation and replication at the level of landscapes is impossible (Phillips 2007). Consequently, models have been used as prime tools to investigate disturbance interactions. Theoretical models can, for instance, be used to understand disturbance linkages from a population dynamics perspective (Økland and Bjørnstad 2006), while simulation models can be applied to capture dampening interactions via feedbacks on vegetation structure and composition over extended time horizons (Temperli et al. 2013a). Despite the high potential of modeling for understanding disturbance interaction, disturbance modeling has to a large degree focused on individual disturbance agents to date. A recent review showed that only a small number of approaches are currently able to address the complexity of interacting disturbances (Seidl et al. 2011). A particular challenge here is that in order to explore the climate sensitivity of disturbance interactions with models, process-based modeling approaches are needed. In order for them to deliver robust results these models need to simulate disturbance interactions as an emergent property of the underlying system dynamics, and account for climate effects based on first principles of ecology (Gustafson 2013). Examples for process-based models of prominent forest disturbance agents are given by Powell and Bentz (2014), Perez and Dragicevic (2012), Hale et al. (2015), and Seidl et al. (2014a).

Here we focus on the interaction between wind and bark beetle disturbances, which are the most important abiotic and biotic disturbance agents in Europe’s forest ecosystems (Seidl et al. 2014b). A strong interaction effect has been documented between these two agents in Norway spruce (*Picea abies* (L.) Karst.) forests previously (Eriksson et al. 2005; Økland and Bjørnstad 2006; Stadelmann et al. 2014): Trees freshly broken or uprooted by wind are preferred breeding material for the European spruce bark beetle (*Ips typographus* L., Coleoptera: Curculionidae), which is the most important bark beetle species in terms of tree mortality in Europe. These windfelled trees are virtually defenseless and allow the beetles to build up the local population densities that are required to successfully attack also healthy trees and form eruptive outbreaks (Wermelinger 2004; Kausrud et al. 2012). Although large outbreaks of the European spruce bark beetle are also possible in the absence of preceding wind disturbance (Kautz et al. 2011; Hlásny and Turčáni 2013) a major share of recent bark beetle damage in Europe’s forest accrued in the immediate temporal and spatial proximity of large wind disturbance events (Kärvemo et al. 2014; Stadelmann et al. 2014). Previous analyses focusing on the two disturbance agents independently have suggested that both wind and bark beetle disturbances could increase in Europe under climate change (Jönsson et al. 2009; Seidl et al. 2009; Blennow et al. 2010; Schelhaas et al. 2010). Yet, how the crucial interaction between wind and bark beetles is affected by a changing climate remains poorly understood. This, in part, results from a current lack of models being able to investigate the climate sensitivity of the wind–bark beetle disturbance regime in Europe (but see Jönsson et al. 2012; Temperli et al. 2013a).

Here our objectives were (i) to develop a new process-based model for simulating bark beetle disturbances, integrated in a dynamic forest landscape modeling framework (which already includes a detailed model of wind disturbance), (ii) to test this dynamic landscape and disturbance model against observations of an eight year wind–bark beetle disturbance series at a mountain forest landscape in Austria, and (iii) to investigate the climate sensitivity of the wind–bark beetle disturbance interaction by conducting a factorial simulation experiment under different combinations of climate forcings. Based on previous studies we expected both wind and bark beetle disturbances to be sensitive to a changing climate, with disturbed area hypothesized to increase with temperature, peak wind speed, and water limitation (Jönsson et al. 2009; Peltola et al. 2010; Seidl et al. 2014a; Netherer et al. 2015). However, we additionally hypothesized that disturbance interactions amplify the climate sensitivity of the disturbance regime beyond the additive effect of changes in the individual agents. This hypothesis is based on an expected nonlinear response to increased outbreak initiation through wind disturbance, as a result of a climate-mediated proliferation in beetle population dynamics.

## Methods and materials

### The iLand simulation framework

iLand (the individual-based forest landscape and disturbance model) was developed to simulate the dynamic interactions between climate change, vegetation dynamics, and disturbances (Seidl et al. 2012). It operates at the grain of individual trees, for which it simulates competition for resources spatially explicit in space and time. Landscape-scale processes such as the dispersal of seeds or the spread of disturbances are simulated explicitly over extents of several tens of thousands of hectares. iLand is a process-based model, in which stand-level gross primary production is simulated based on a light use efficiency approach, and combined with ecological field theory for determining the resource availability for every tree (Seidl et al. 2012). The effects of environmental constraints on vegetation development are accounted for at daily time steps. Individual tree mortality is calculated based on carbon starvation, and regeneration of new seedlings depends on the local presence of seeds, light, and a favorable abiotic environment. iLand tracks ecosystem carbon stocks and fluxes, and is able to simulate detailed forest management interventions via an agent-based management model (Rammer and Seidl 2015). The model has previously been parameterized and tested for ecosystems in Central and Northern Europe as well as Western North America, and was successfully applied to simulate decadal to millennial scale forest dynamics for landscapes between 2500 and 25,000 hectares.

iLand is particularly suited to study disturbance interactions as it operates at a fine spatial and temporal grain while being able to simulate disturbance processes spatially explicit at the landscape scale. Wildfire and wind disturbances have been included in the model in previous efforts. Wind damage is modeled at the level of individual trees with wind disturbance events being simulated iteratively, dynamically accounting for changes in forest structure during the course of a storm (Seidl et al. 2014a). Both upwind gap size and local shelter from neighboring trees are considered explicitly, and critical wind speeds for uprooting and stem breakage are distinguished in the model. Tree response to wind is derived from empirically parameterized turning moment coefficients (Hale et al. 2010). Besides the dynamically simulated forest structure and composition, major drivers of wind disturbance are wind speed and direction, storm duration, and soil frost (the latter influencing the anchorage of a tree). For details on simulating wind disturbance in iLand as well as a sensitivity analysis and thorough test against independent data we refer to Seidl et al. (2014a).

### Bark beetle modeling

The bark beetle component was newly developed for the current study, and builds on recent advances in process-based modeling of *I. typographus* disturbance dynamics (Seidl et al. 2007; Fahse and Heurich 2011; Kautz et al. 2011; Jönsson et al. 2012; Kautz et al. 2014). It explicitly considers bark beetle phenology and development, spatially explicit dispersal of beetles, colonization and tree defense, as well as temperature-related overwintering success. The design of the models follows recent findings on multi-scale drivers of bark beetle outbreaks (see Raffa et al. 2008; Seidl et al. 2016a), considering processes at the tree (defense, susceptibility), stand (thermal requirements and beetle phenology), landscape (host distribution, beetle dispersal) and regional (climate variation and extremes as triggers of outbreaks) scale. The new module is here described and parameterized for the *P. abies*–*I. typographus* system, but could also be adapted for other bark beetle species in the future, as the design is general and process-based. The following paragraphs give a brief overview of the model, with a more detailed description given in Appendix A of the Online Supplementary Material.

Potential host trees for the beetle are defined as *P. abies* individuals exceeding a threshold diameter at breast height (dbh) of 15 cm (Seidl et al. 2007). An outbreak is initiated either through a climate-sensitive background probability or through a wind disturbance event being simulated in the model. The probability for windthrown or -broken trees to being colonized by bark beetles is set to 0.3, based on previous empirical analyses (Eriksson et al. 2005, 2008). Once an outbreak is initiated, beetle development is simulated by means of a phenology-based model of bark beetle development (Baier et al. 2007; Seidl et al. 2007). If the thermal conditions allow the completion of the beetles’ developmental cycle, beetles disperse from the brood tree in a two-step approach: First, beetle flight follows a symmetrical dispersal kernel (Fahse and Heurich 2011; Kautz et al. 2011). The direction of dispersal is randomly chosen and the distance is determined from the probabilistic kernel function. In a second step the thus determined approximate landing position is further modified by the beetle actively searching for potential hosts in its vicinity. The perceptual range of the beetles for this search was previously estimated to be in the range of 15 m (Fahse and Heurich 2011; Kautz et al. 2014), and here the eight-cell neighborhood in a 10 m grid is used. Within this search radius, beetles prefer wind-disturbed potential host trees over healthy host trees if the former are available. Rather than simulating the dispersal of individual beetles explicitly the model tracks beetle cohorts, with a cohort being defined as the minimum number of beetles that are needed to successfully colonize a tree (estimated to 30 beetles in the case of *I. typographus*, Kautz et al. 2014). Every brood tree disperses a number of such beetle cohorts determined by the reproductive rate of the beetle, estimated to range between 4 and 24 by Wermelinger and Seifert (1999), and set to 20 in this study.

A beetle cohort attacking a tree has to overcome the trees’ defense system, which is here modeled as a function of the dynamically simulated nonstructural carbohydrate reserves of the attacked tree. We follow Kautz et al. (2014) in assuming that a healthy, vigorous tree (i.e., a host tree at its maximum defense capacity) requires 6.7 times more beetles attacking it in order to being successfully colonized compared to a stressed tree. During a dispersal wave multiple beetle cohorts can attack a potential host tree. Furthermore, if the climate is conducive for the beetle to develop additional generations per year, the dispersal and colonization routine described above is repeated within the same year. Only the last beetle generation developing in a year is assumed to overwinter. Of that generation, all immature beetles experience complete winter mortality (Faccoli 2002; Jönsson et al. 2012). For mature beetles, a fixed rate of beetles is assumed to die over winter (set to 40 %, Jönsson et al. 2012), with additional mortality occurring if a frost threshold of −15 °C is exceeded (Koštál et al. 2011). Antagonists are another important source of beetle mortality (Wermelinger 2002), yet antagonist population dynamics is not explicitly simulated in iLand (but see Fahse and Heurich 2011). Beetle mortality from antagonists and density-dependent mortality in later stages of an outbreak is modeled phenomenologically, as a function of the time elapsed from the initiation of the outbreak, and is parameterized to mimic the outbreak durations typically observed in the *P. abies*–*I. typographus* system (Kautz et al. 2011). Bark beetle management can be accounted for in the model via removing infested trees before the brood can emerge or via trap trees (i.e., felling potential host trees to attract beetles, and removing them before their offspring are fully developed). Also salvage logging can be simulated within the iLand management module (Rammer and Seidl 2015) in order to reduce the risk for bark beetle outbreaks. More details on the bark beetle module can be found in Appendix A of the Online Supplementary Material. Furthermore, we conducted a sensitivity analysis of crucial model parameters, specifically beetle reproduction rate, infestation probability of windthrown trees, maximum outbreak duration, minimum host tree diameter, and beetle dispersal, to test their influence on the simulated area disturbed (Online Appendix B).

### Study landscape and recent disturbance history

As study landscape we here focused on the Kalkalpen national park (KANP), a 20,856 ha landscape situated in the northern front range of the Alps in Austria (N47.47°, E14.22°). The KANP is characterized by steep mountainous terrain covering an elevation range from 385 m to 1963 m asl. Mean annual temperature decreases strongly with elevation, while mean annual precipitation sum increases (Table 1). On average 58 % of the annual precipitation sum accrues from April to September, but the generally shallow soils can result in water limitations during dry periods in summer. Predominant soil types are Rendzic Leptosols and Cromic Cambisols over calcareous bedrock. The natural vegetation is dominated by European beech (*Fagus sylvatica* L.) in low and mid-elevation areas, with mixed forests of beech, Norway spruce and Silver fir (*Abies alba* Mill.) forming the montane vegetation belt, and subalpine Norway spruce forests dominating the highest reaches of the park. Current vegetation still reflects past management practices, with an overabundance of Norway spruce (44 % of the basal area on the landscape) relative to the natural vegetation composition. The national park was established in 1997, and its 13,865 ha of protected forest make it the largest contiguously forested conservation area in the Eastern Alps. For detailed climate and soil data at KANP as well as for an evaluation of iLand at the park we refer to Thom et al. (2016).

**Table 1:** Characteristics of the study landscape Nationalpark Kalkalpen

Description	Elevation		
	<800 m	800–1200 m	>1200 m
Mean annual temperature (°C)	10.4	8.9	7.5
Mean temperature April–September (°C)	15.0	14.6	13.0
Mean annual precipitation sum (mm)	1249	1339	1471
Mean precipitation sum April–September (mm)	756	867	908
Mean basal area (m^2^ ha^−1^)	25.6	26.5	21.3
Share of Norway spruce on total basal area (%)	33.3	39.6	55.6

Climate variables are given for the period 2007–2014, while information on stand structure and composition pertains to the year 2007

Here, we focus on the disturbance dynamics at KANP from 2007 to 2014. We calibrated and tested the newly developed bark beetle simulation module against KANP data, and investigated the climate sensitivity of disturbance interactions using the 2007 to 2014 series at KANP as a starting point. Data on disturbed area were extracted from satellite-based remote sensing at 30 m horizontal resolution (Hansen et al. 2013). For the years with both wind and bark beetle disturbance (2007 and 2008) an attribution to the respective agent was achieved using estimates of KANP staff on disturbed timber volume by agent. This attribution of the satellite-derived disturbance data was based on terrestrially observed damage shares and was not spatially explicit. The recent disturbance dynamics at KANP was dominated by two wind events followed by an outbreak of *I. typographus*. In the night from January 18th–19th 2007 the storm “Kyrill” hit the area from west-southwesterly direction, with peak mean hourly wind speeds of 12.8 m s^−1^, and a storm duration of approximately 8 h. Kyrill caused extensive forest damage throughout the region, with approximately 113.3 ha disturbed in the KANP. Only one year after Kyrill another sequence of strong wind events affected the area: On January 26th 2008 the storm “Paula” (peak mean hourly wind speeds of 11.5 m s^−1^, approximate storm duration of 3 h) affected the area from west-northwesterly direction. Only a few weeks later, the storm “Emma” (March 1st 2008) caused additional damage in the area (main wind direction: west, peak mean hourly wind speed: 12.8 m s^−1^, approximate storm duration: 1 h). Together, Paula and Emma disturbed 56.0 ha of forest at the KANP in 2008. Following these wind events a major bark beetle outbreak developed in the area. Along the national park border the outbreak was managed via trap trees and sanitation felling, particularly from the year 2010 onwards. For this reason we here focus on the bark beetle development at KANPs core zone, a forest area of 9336 ha under strict protection and not subject to interventions from management. The outbreak peaked in 2011 at an annual area newly infested of 112.6 ha. After 2011, a sharp decline in new infestations was observed, with only 3.0 ha being affected by bark beetle from 2012 to 2014. Overall, the wind–bark beetle series analyzed here disturbed a total of 530.2 ha between 2007 and 2014 (wind: 169.3 ha, bark beetles: 360.9 ha), which corresponds to 3.8 % of the forested area of the KANP.

### Analyses

A main objective here was to evaluate our disturbance modeling against empirical data. To that end we ran simulations for the KANP under observed climate conditions from 2007 to 2014 and compared results to disturbance data derived from remote sensing. Only simulated disturbed areas consisting of more than four adjacent 10 m grid cells were considered in the analysis, in order to match the grain of the simulation data to the reference data from remote sensing (Hansen et al. 2013). No site-specific calibration was conducted for the process-based wind disturbance module of iLand, which had been parameterized in a previous study (Seidl et al. 2014a). The newly developed bark beetle module was parameterized based on detailed process information obtained from the literature (Online Appendix B). Due to the large sensitivity of the model to the reproduction rate of the beetle and the particularly wide range of reported values in the literature (Online Appendix B) we used this parameter for calibration against the total observed bark beetle damage 2007–2014 (see also Temperli et al. 2013a). The temporal development, climate sensitivity, spatial pattern and spread as well as size and severity of the simulated bark beetle outbreak were not calibrated, and are an emergent property of the process-based simulation framework.

Subsequently, we conducted an attribution analysis for the wind–bark beetle disturbance series 2007–2014, with the aim to identify how much the interaction effect between the two agents contributed to the overall disturbance. To that end we ran the model for both disturbance agents individually, determining the main effects of wind (*M*_*w*_) and bark beetles (*M*_*b*_). Subsequently, we quantified the interaction effect (*I*_*wb*_) by subtracting the total main effect (*M*_*wb*_ = *M*_*w*_ + *M*_*b*_) from the result of a simulation with full interactions between the two agents. We tested the Null hypothesis of no interaction effects by comparing *I*_*wb*_ against zero using Student’s *t* test.

To subsequently investigate the climate sensitivity of wind–bark beetle interactions we conducted a factorial simulation experiment determining *M*_*wb*_ and *I*_*wb*_ under a range of different climate forcings. Specifically, we studied the effect of a warming of +2 and +4 °C, a precipitation change of −33 and +33 %, and a change in mean hourly peak wind speeds of −10 and +10 %. These changes were applied uniformly in space and time, preserving the spatial differences in variables on the landscape as well as the intra- and interannual variation in climate drivers of the observation period. A sensitivity analysis on the effect of inter-annual climate variability can be found in Online Appendix C. Simulations for all factorial combinations were replicated ten times to account for stochasticity, mainly introduced by a probabilistic derivation of beetle dispersal and colonization, as well as winter survival of beetles. The climate sensitivity of cumulative main and interaction effects (i.e., the sum totals of *M*_*wb*_ and *I*_*wb*_ over the 8 year study period) were analyzed by means of analysis of variance and multiple linear regression analysis, using a square root transformation of the dependent variable. Under the Null hypothesis that climate change does not influence the interaction strength between disturbance agents we would expect the change rates of *I*_*wb*_ with climate to be not significantly different from zero in this analysis.

## Results

### Evaluation of disturbance simulations

**Fig. 1:**
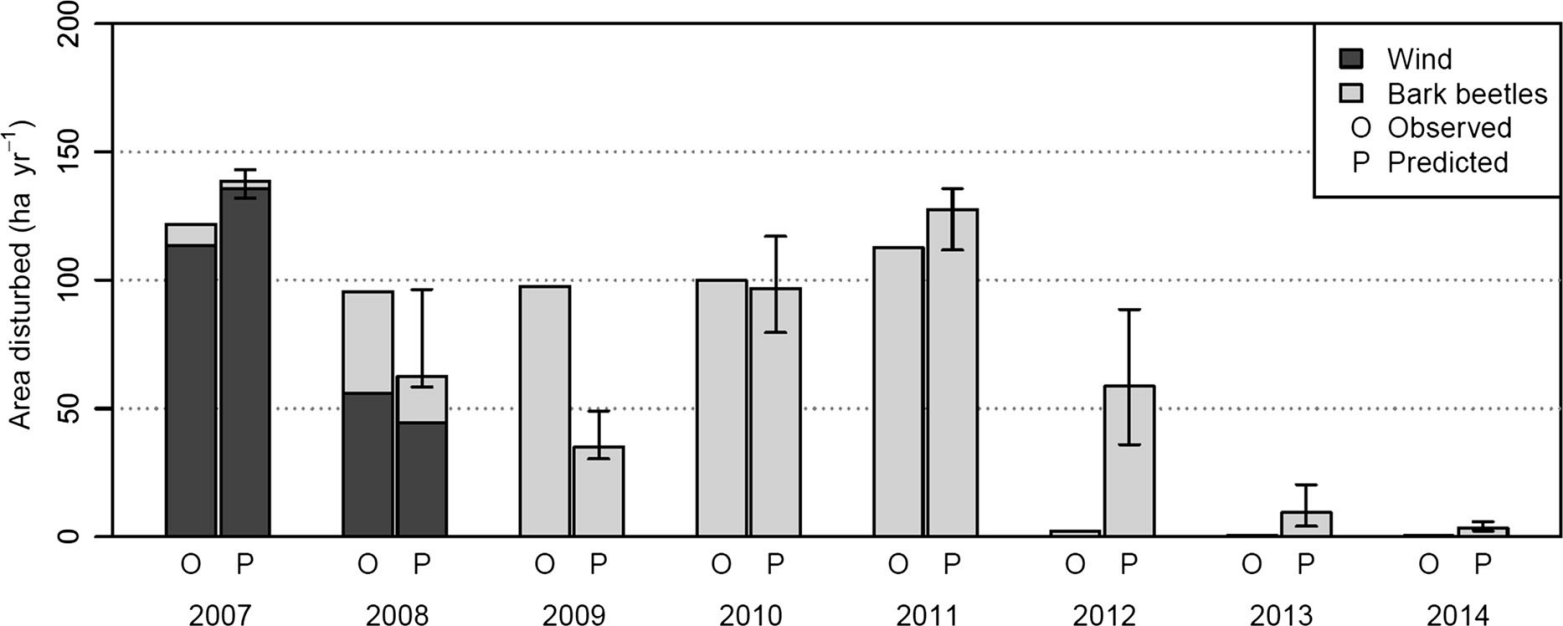
Observed and predicted disturbances by wind and bark beetles at the Kalkalpen National Park in the northern front range of the Alps in Austria. Predictions are the median over ten replicated model runs, with *whiskers* indicating the range over the replicates

Overall, iLand was well able to reproduce the disturbance dynamics at the KANP from 2007 to 2014 (Fig. 1). The overall area affected by the three wind events of 2007 and 2008 was well reproduced by iLand (observed: 169.3 ha, simulated mean over all replicates: 182.3 ha, min: 169.9 ha, max: 218.7 ha). The area affected by storm Kyrill (2007) was moderately overestimated by the model (+18.4 %), while the effect of Paula and Emma (2008) was underestimated by approximately the same amount (−14.0 %). Nonetheless, 2007 was simulated as the considerably more extensive event compared to 2008, which corresponds well to observations.

The calibrated overall amount of bark beetle damage 2007–2014 matched the observations well (observed: 360.9 ha, simulated mean: 352.7 ha, min: 302.3 ha, max: 395.6 ha). Also the observed temporal pattern of newly infested area was reproduced by the model, with simulated bark beetle damage peaking 3 years after the last storm (Fig. 1) at a level of 124.9 ha year^−1^ (observed: 112.6 ha year^−1^). However, bark beetle disturbance spread slower than observed in the initial phases of the outbreak (years 2008 and 2009). Furthermore, although the model was able to simulate a decrease in the newly infested area after the outbreak peak in 2011, it did not fully capture the abrupt breakdown of the infestation recorded in the remote sensing data.

In addition to temporal disturbance dynamics also the spatial distribution of disturbed area on the landscape was successfully reproduced by the model. The largest share of the disturbed area was simulated for the mid-elevation zone of the KANP, which is well in line with observations (Table 2). Only 3.8 % of the 531.7 ha disturbed were below 600 m or above 1500 m in elevation in the simulations (observation: 5.6 %). With regard to the patch size distribution, the overwhelming majority of patches were <1 ha in size (observed: 81.3 %, predicted: 95.5 %). When analyzing the area-weighted patch size, however, it gets clear that a small number of large patches made a considerable contribution to the overall area disturbed. This pattern was poorly reproduced by the model, where the majority of simulated disturbance occurred in small patches (Table 2).

**Table 2:** Distribution of disturbed area 2007–2014 over elevation and patch size

	Observed % of disturbed area	Predicted % of disturbed area
Elevation		
<600 m	0.6	1.5 (1.3–2.5)
600–900 m	14.6	26.1 (25.0–28.1)
900–1200 m	43.8	48.1 (45.3–49.9)
1200–1500 m	36.0	22.1 (21.0–23.6)
>1500 m	5.0	2.3 (1.7–2.4)
Patch size		
<1 ha	23.9	64.9 (56.9–64.9)
1–2 ha	18.3	15.0 (13.3–17.6)
2–4 ha	14.3	9.1 (9.1–15.1)
4–6 ha	12.7	6.7 (4.3–0.3)
6–8 ha	9.2	1.2 (1.2–5.8)
>8 ha	21.7	3.2 (0.0–3.5)

Predictions are calculated for the run with the median disturbed area out of ten replicated simulations (range in parenthesis)

### Attribution of disturbance

**Fig. 2:**
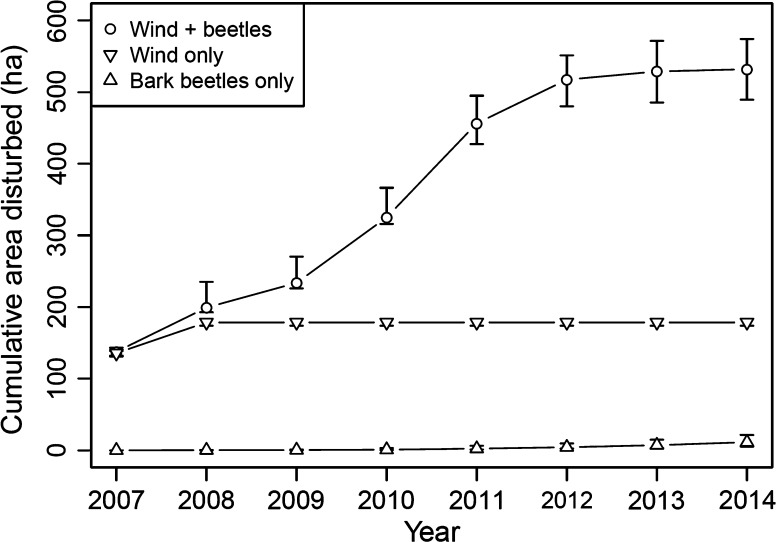
Attribution of the simulated disturbance dynamics into the main effects of wind and bark beetles, and the interaction effect of these two agents. *Whiskers* indicate the range over the ten simulated replicates

Reanalyzing the 2007–2014 disturbance series by means of a factorial simulation experiment revealed that a large interaction effect contributed significantly (p < 0.001) to the overall disturbed area. *I*_*wb*_ was estimated to amount to 342.1 ha over the 8 year period, contributing 64.3 % to the overall area affected by disturbance. Without the inciting effect of wind disturbance the simulated bark beetle disturbance (*M*_*b*_) remained at a low level of 11.3 ha (Fig. 2).

### Climate sensitivity of wind–bark beetle interactions

**Fig. 3:**
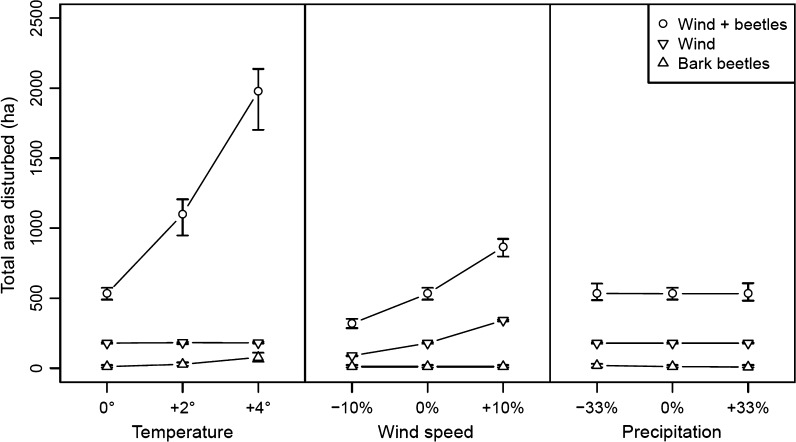
The sensitivity of wind and bark beetle disturbance to changes in temperature, peak wind speed, and precipitation. Values are the total area disturbed at the end of the 8 year study period. For each panel, the other climate variables were kept unchanged at their default values. *Whiskers* indicate the range over ten simulated replicates

**Fig. 4:**
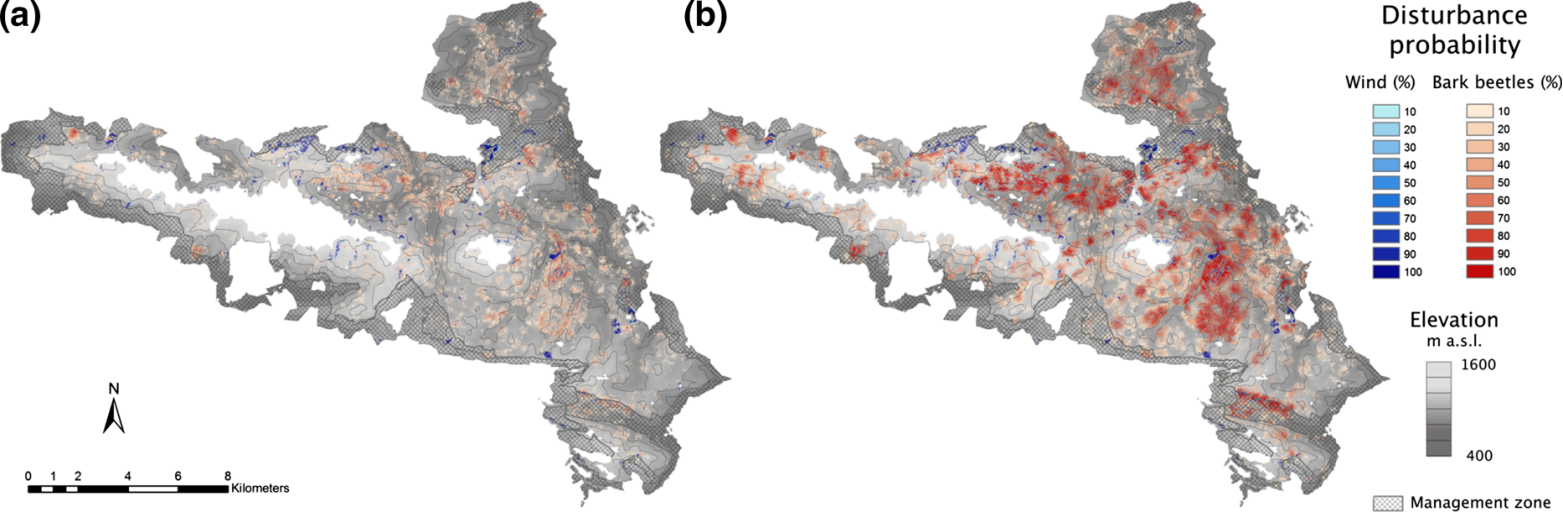
Map of the Kalkalpen National Park and the simulated cumulative wind and bark beetle disturbance 2007–2014 **a** under observed climatic conditions, and **b** assuming an increase in temperature of +4 °C. Disturbance probability was calculated as the number of times a 10 m pixel was disturbed over all simulated replicates divided by the number of replicates simulated (n = 10). Please note that the analyses on bark beetle disturbances presented in this contribution disregard the management zone along the park boundaries

Of the three climate variables investigated wind speed had the strongest influence on the main disturbance effect *M*_*wb*_, accounting for 88.3 % of the overall variance in the simulated area disturbed without interaction (Table 3). With all other variables remaining at their current level a 10 % increase in peak wind speed increased the area disturbed by wind (*M*_*w*_) by +384 % (Fig. 3). However, also the influence of temperature and precipitation on the main disturbance effect were significant, with a +4 °C warming increasing the area disturbed by bark beetles (*M*_*b*_) by +684 %. Interactions were found to have an even higher sensitivity to climate than main effects. For changes in temperature, for instance, the climate sensitivity of interaction effects exceeded the climate sensitivity of main effects by a factor of 10 (Table 3). The combination of wind as a triggering event with warming-related increases in beetle development rates in higher elevation areas and the abundance of potential host trees in these mid- to high elevation parts of the landscape (see Online Appendix C) resulted in a strongly amplified bark beetle outbreak (Fig. 4). A +4 °C warming alone increased the disturbed area attributed to an interaction effect more than four-fold, and increased the area-weighted mean patch size of disturbance from 1.57 ha to 29.74 ha. The most extreme scenario combination studied, consisting of a +4 °C and a +10 % increase in peak wind speeds at a simultaneous decrease in precipitation by −33 %, resulted in a total area disturbed of 2563.3 ha ± 114.0 ha (+482 % relative to baseline conditions), whereof 2106.5 ha ± 106.1 ha (82.2 %) could be attributed to interaction effects. In other words: If unfolding under such extreme conditions, the 2007–2014 disturbance series would have affected 27.5 % of the landscape.

**Table 3:** Sensitivities of main effect (i.e., the disturbance accrued through wind and bark beetle disturbances acting in isolation) and interaction effect (i.e., the additional area disturbed through the interaction between wind and bark beetles) to changes in climate variables

Climate parameter	Main effect *M*_*wb*_ (Intercept: 14.14)		Interaction effect *I*_*wb*_ (Intercept: 18.69)	
	Effect size	Contribution to total variance (%)	Effect size	Contribution to total variance (%)
Temperature change (°C)	+0.56	6.6	+5.60	82.7
Wind speed change (%)	+0.41	88.3	+0.48	15.2
Precipitation change (%)	−0.02	1.4	<0.01^*ns*^	<0.1

Effect size was determined by means of linear regression, and coefficients are significant at α = 0.05 unless otherwise noted (*ns* not significant). The response variable was the square root transformed cumulative area disturbed at the end of the 8 year study period. The influence of the individual climate variables on disturbed area was determined via the contribution of the variable to the total variance by means of analysis of variance

## Discussion and conclusion

### Disturbance modeling

We here presented a process-based module of bark beetle disturbances integrated into a dynamic landscape and disturbance modeling framework, in order to study the climate sensitivity of disturbance interactions. The development of the bark beetle module aimed to combine recent advances in process-understanding (Fahse and Heurich 2011; Kautz et al. 2014) with the main strength of dynamic vegetation models, i.e., to dynamically address disturbance–vegetation feedbacks (see Seidl et al. 2011). In the context of existing approaches the bark beetle disturbance module presented here features high temporal resolution, considering the weather-dependent intra- and interannual dynamics of beetle development and spread explicitly, rather than operating at decadal time-steps (cf. Temperli et al. 2013a). Furthermore, the spatial resolution of the newly developed model is high relative to previous approaches (cf. Jönsson et al. 2012), and spatial dependencies are considered explicitly (i.e., beetle spread is simulated at 10 m horizontal grid cells, and successful colonization depends on the beetles being able to find suitable hosts within their domain of activity). This allows the simulation of landscape patterns related to beetle attacks as emergent property, and facilitates a process-based simulation of interactions with wind disturbance, as defenseless downed host trees are the central element of the wind–bark beetle interaction in *P. abies*–*I. typographus* systems (Wermelinger 2004; Kausrud et al. 2012).

However, relative to more detailed models of bark beetle development, simplifications were made to achieve computational scalability and parsimony in model structure and parameters. We, for instance, did not model individual beetle communication and decisions explicitly (Bone and Altaweel 2014; Kautz et al. 2014), but rather addressed this process by aggregating beetles into cohorts approximating collective behavior. Furthermore, antagonists were not simulated explicitly (but see Fahse and Heurich 2011), inter alia due to the high diversity in the antagonist community (Wermelinger 2002), which complicates a process-based modeling approach. This is an important limitation in the context of projecting the effects of potential future climate change, as it remains widely unclear how the antagonist community will respond to such climatic changes (Netherer and Schopf 2010). Despite these simplifications, an initial evaluation of the integrated modeling framework showed that the dynamics of an 8 year disturbance series at the landscape scale were satisfactorily reproduced by the model with regard to area, sequence, pattern, and distribution of disturbance on the landscape.

Uncertainties remain, amongst other things, with regard to model parameterization (see also Online Appendix B). For instance, we here employed a single threshold diameter to determine whether a tree is suitable to be colonized by *I. typographus*, and used a value at the lower end of the spectrum for the simulations presented here. This approach corresponds to the observation that beetles are not selective under outbreak conditions, and attack potential hosts regardless of their diameter (Sproull et al. 2015). If population density is low, however, beetles might preferably attack larger diameter trees as they provide better resources for reproduction, an effect that is currently not considered in our model.

Further uncertainties exist with regard to model–data comparisons in the context of disturbance modeling. Despite the growing capacity to determine forest disturbances from remote sensing products (McDowell et al. 2015) the 30 m resolution of the reference dataset used here (Hansen et al. 2013) foregoes an evaluation of, e.g., patch shape and complexity metrics. Furthermore, attribution of remotely sensed disturbed areas to disturbance agents remains challenging. Particularly in the management zone of the KANP it was not possible to clearly separate proactive beetle control measures (e.g., the felling of trap trees) from natural disturbance, which is why we excluded this zone from our analyses of bark beetle dynamics. Another aspect complicating a comparison between simulations and observations is the remaining uncertainty regarding the initial conditions of the system. We did not have information on prior bark beetle activity available, which is why we started our simulations with a landscape completely devoid of bark beetles. This clearly is an unrealistic assumption, and possibly led to an underestimation of *M*_*b*_ in our simulations. Furthermore, also the initial vegetation structure and composition likely introduces uncertainty in the simulations (see Temperli et al. 2013b). We here used a data-intensive combination of remote sensing products, field inventories, and simulation to determine forest structure and composition at KANP in 2007 (see Thom et al. (2016) for details). Yet the moderate overestimation of the storm Kyrill in the first simulation year is likely the result of the initialized forest featuring unrealistic structures with regard to edges or exposed trees. In the context of a long-term simulation the effects of such artifacts of initialization are expected to strongly decrease, as the simulated vegetation dynamically adapts to the prevailing wind regime. Over longer periods also an interaction effect between bark beetles and wind could be hypothesized, with beetles creating gaps and edges which in turn increase the susceptibility to wind disturbance. However, due to the specific sequence of the disturbance series analyzed here (storm events in the first 2 years of the study period), the current analysis addressed solely interactions of wind influencing bark beetle disturbance (and not vice versa).

### Disturbance interactions under climate change

As hypothesized, we found a strong linked interaction effect between wind and bark beetle disturbances (see also Eriksson et al. 2005; Økland and Bjørnstad 2006; Stadelmann et al. 2014). Furthermore, our results underline the high climate sensitivity of the wind–bark beetle disturbance regime, a finding that is well in line with previous studies (Temperli et al. 2013a). We here showed that linked interactions amplify the climate change sensitivity of forest disturbance dynamics. Our analysis revealed that both changes in temperature and peak wind speed have a strong effect on the studied disturbance regime. Interestingly, precipitation changes had a considerably smaller effect on disturbance activity, which is at first glance contradictory to empirical findings (Netherer et al. 2015). However, the ±33 % variation in precipitation investigated here might not have been enough to trigger severe water limitation and subsequently reduced tree defense, given the relatively high base level of growing season precipitation in the study area (see Table 1). Also, the study period 2007–2014 did not contain a strong drought year, compared, for instance, to the conditions of the drought of 2003 (Rouault et al. 2006). Furthermore, the sensitivity of the *P. abies*–*I. typographus* system to precipitation was recently found to be contingent on outbreak stage (Seidl et al. 2016a). Studying a single outbreak event as done here might thus not be sufficient to capture the sensitivity of the system to drought.

In addition to a strong and highly climate sensitive interaction effect between wind and bark beetles we also found evidence of cascading effects of climatic changes through disturbance interactions (Buma 2015): Despite the fact that wind speed did not influence bark beetles directly in our model, the area disturbed by beetles responded positively to elevated peak wind speeds, as a result of an increased number of potential starting points for outbreaks (see central panel in Fig. 3). Interactions thus have the potential for strong and nonlinear amplification of the forest disturbance regime.

In this context an important issue is whether the high sensitivity of the wind–bark beetle disturbance regime determined here by means of modeling is ecologically meaningful. In this regard it is interesting to note that wind and bark beetle disturbances have already increased more than three-fold in Europe over the last 40 years, and that scenario analyses suggest a strong further increase for the 21st century in response to warming (Jönsson et al. 2009; Seidl et al. 2009; Temperli et al. 2013a; Seidl et al. 2014b). Furthermore, the outbreak rates predicted under the most extreme scenario combinations studied here are comparable to a recent *I. typographus* outbreak at the Bavarian Forest National Park (located approximately 150 km to the northwest of our study area), where bark beetles disturbed 6500 ha in two outbreak waves over 25 years (Kautz et al. 2011; Seidl et al. 2016a). Despite the fact that our findings are well in line with these observations it is likely that long-term disturbance–vegetation feedbacks will dampen the climate sensitivity of disturbance regimes. By the time climate warming might reach the levels studied here also the prevailing forest composition and structure might have changed, potentially resulting in a reduced susceptibility of the landscape (e.g., when warming facilitates European beech over Norway spruce). In addition, small wind events or beetle outbreaks might catalyze such changes, reducing the propensity for large and landscape-wide events such as simulated here (Temperli et al. 2013a).

Nonetheless, our analysis suggests that increasing disturbance activity is likely in Central Europe’s mountain forests in a warming climate. Increasing disturbances do, however, not necessarily threaten conservation goals in protected areas such as the KANP, as disturbance activity fosters tree species diversity and ecosystem processes (Silva Pedro et al. 2016). However, if disturbance interactions lead to much larger patch sizes, species particularly vulnerable to large, open areas might increasingly suffer (Thom et al. 2016). Furthermore, interactions as the one studied here also hold the potential for compounding effects, potentially eroding the resilience of forests and resulting in ecological surprises (Paine et al. 1998; Seidl et al. 2016b). Such potential impacts of amplifying disturbance interactions on ecosystems underline the importance of considering disturbance agents not in isolation but in the dynamic context of their disturbance regime. Our analysis suggests that addressing disturbance agents individually but neglecting their interactions could lead to a significant underestimation of the climate sensitivity of disturbance regimes. An integrated consideration of climate change, disturbances, and forest dynamics is thus needed to assess potential future trajectories of forest landscapes.

## Electronic supplementary material

Below is the link to the electronic supplementary material.

Supplementary material 1 (DOCX 225 kb)

## References

[CR1] Baier P, Pennerstorfer J, Schopf A (2007). PHENIPS—a comprehensive phenology model of *Ips typographus* (L.) (Col., *Scolytinae*) as a tool for hazard rating of bark beetle infestation. For Ecol Manag.

[CR2] Bebi P, Kulakowski D, Veblen TT (2003). Interactions between fire and spruce beetles in a subalpine Rocky Mountain forest landscape. Ecology.

[CR3] Blennow K, Andersson M, Sallnäs O, Olofsson E (2010). Climate change and the probability of wind damage in two Swedish forests. For Ecol Manag.

[CR4] Bone C, Altaweel M (2014). Modeling micro-scale ecological processes and emergent patterns of mountain pine beetle epidemics. Ecol Model.

[CR5] Buma B (2015). Disturbance interactions: characterization, prediction, and the potential for cascading effects. Ecosphere.

[CR6] Buma B, Wessman CA (2011). Disturbance interactions can impact resilience mechanisms of forests. Ecosphere.

[CR7] Eriksson M, Neuvonen S, Roininen H (2008). *Ips typographus* (L.) attack on patches of felled trees: “Wind-felled” vs. cut trees and the risk of subsequent mortality. For Ecol Manag.

[CR8] Eriksson M, Pouttu A, Roininen H (2005). The influence of windthrow area and timber characteristics on colonization of wind-felled spruces by *Ips typographus* (L.). For Ecol Manag.

[CR9] Faccoli M (2002). Winter mortality in sub-corticolous populations of *Ips typographus* (Coleoptera, Scolytidae) and its parasitoids in the south-eastern Alps. Anzeiger fur Schädlingskd.

[CR10] Fahse L, Heurich M (2011). Simulation and analysis of outbreaks of bark beetle infestations and their management at the stand level. Ecol Model.

[CR11] Gustafson EJ (2013). When relationships estimated in the past cannot be used to predict the future: using mechanistic models to predict landscape ecological dynamics in a changing world. Landscape Ecol.

[CR12] Hale SA, Gardiner B, Peace A, Nicoll B, Taylor P, Pizzirani S (2015). Comparison and validation of three versions of a forest wind risk model. Environ Model Softw.

[CR13] Hale SE, Gardiner BA, Wellpott A, Nicoll BC, Achim A (2010). Wind loading of trees: influence of tree size and competition. Eur J For Res.

[CR14] Hansen MC, Potapov PV, Moore R, Hancher M, Turubanova SA, Tyukavina A, Thau D, Stehman SV, Goetz SJ, Loveland LR, Kommareddy A (2013). High-resolution global maps of 21st-century forest cover change. Science.

[CR15] Hart SJ, Schoennagel T, Veblen TT, Chapman TB (2015). Area burned in the western United States is unaffected by recent mountain pine beetle outbreaks. Proc Natl Acad Sci.

[CR16] Harvey BJ, Donato DC, Turner MG (2014). Recent mountain pine beetle outbreaks, wildfire severity, and postfire tree regeneration in the US Northern Rockies. Proc Natl Acad Sci.

[CR17] Hlásny T, Turčáni M (2013). Persisting bark beetle outbreak indicates the unsustainability of secondary Norway spruce forests: case study from Central Europe. Ann For Sci.

[CR18] Jönsson AM, Appelberg G, Harding S, Bärring L (2009). Spatio-temporal impact of climate change on the activity and voltinism of the spruce bark beetle, *Ips typographus*. Glob Change Biol.

[CR19] Jönsson AM, Schroeder LM, Lagergren F, Anderbrant O, Smith B (2012). Guess the impact of *Ips typographus*—an ecosystem modelling approach for simulating spruce bark beetle outbreaks. Agric For Meteorol.

[CR20] Kärvemo S, Rogell B, Schroeder M (2014). Dynamics of spruce bark beetle infestation spots: importance of local population size and landscape characteristics after a storm disturbance. For Ecol Manag.

[CR21] Kausrud K, Økland B, Skarpaas O, Grégoire JC, Erbilgin N, Stenseth NC (2012). Population dynamics in changing environments: the case of an eruptive forest pest species. Biol Rev.

[CR22] Kautz M, Dworschak K, Gruppe A, Schopf R (2011). Quantifying spatio-temporal dispersion of bark beetle infestations in epidemic and non-epidemic conditions. For Ecol Manag.

[CR23] Kautz M, Schopf R, Imron MA (2014). Individual traits as drivers of spatial dispersal and infestation patterns in a host–bark beetle system. Ecol Model.

[CR24] Koštál V, Doležal P, Rozsypal J, Moravcová M, Zahradníčková H, Šimek P (2011). Physiological and biochemical analysis of overwintering and cold tolerance in two Central European populations of the spruce bark beetle, *Ips typographus*. J Insect Physiol.

[CR25] Kulakowski D, Jarvis D, Veblen TT, Smith J (2012). Stand-replacing fires reduce susceptibility of lodgepole pine to mountain pine beetle outbreaks in Colorado. J Biogeogr.

[CR26] McDowell NG, Coops NC, Beck PSA, Chambers JQ, Gangodagamage C, Hicke JA, Huang CY, Kennedy R, Krofcheck DJ, Litvak M, Meddens AJ (2015). Global satellite monitoring of climate-induced vegetation disturbances. Trends Plant Sci.

[CR27] Netherer S, Matthews B, Katzensteiner K, Blackwell E, Henschke P, Hietz P, Pennerstorfer J, Rosner S, Kikuta S, Schume H, Schopf A (2015). Do water-limiting conditions predispose Norway spruce to bark beetle attack?. New Phytol.

[CR28] Netherer S, Schopf A (2010). Potential effects of climate change on insect herbivores in European forests—general aspects and the pine processionary moth as specific example. For Ecol Manag.

[CR29] Økland B, Bjørnstad ON (2006). A resource-depletion model of forest insect outbreaks. Ecology.

[CR30] Paine RT, Tegner MJ, Johnson EA (1998). Compounded perturbations yield ecological surprises. Ecosystems.

[CR31] Peltola H, Ikonen V-P, Gregow H, Strandman H, Kilpeläinen A, Venäläinen A, Kellomäki S (2010). Impacts of climate change on timber production and regional risks of wind-induced damage to forests in Finland. For Ecol Manag.

[CR32] Perez L, Dragicevic S (2012). Landscape-level simulation of forest insect disturbance: coupling swarm intelligent agents with GIS-based cellular automata model. Ecol Model.

[CR33] Phillips JD (2007). The perfect landscape. Geomorphology.

[CR34] Pickett ST, White PS (1985). The ecology of natural disturbance and patch dynamics.

[CR35] Powell JA, Bentz BJ (2014). Phenology and density-dependent dispersal predict patterns of mountain pine beetle (*Dendroctonus ponderosae*) impact. Ecol Model.

[CR36] Raffa K, Aukema B, Bentz B, Carroll AL, Hicke JA, Turner MG, Romme WH (2008). Cross-scale drivers of natural disturbances prone to anthropogenic amplification: the dynamics of bark beetle eruptions. Bioscience.

[CR37] Rammer W, Seidl R (2015). Coupling human and natural systems: simulating adaptive management agents in dynamically changing forest landscapes. Glob Environ Change.

[CR38] Rouault G, Candau J-N, Lieutier F, Nageleisen LM, Martin JC, Warzée N (2006). Effects of drought and heat on forest insect populations in relation to the 2003 drought in Western Europe. Ann For Sci.

[CR39] Schelhaas M-J, Hengeveld G, Moriondo M, Reinds GJ, Kundzewicz ZW, Ter Maat H, Bindi M (2010). Assessing risk and adaptation options to fires and windstorms in European forestry. Mitig Adapt Strateg Glob Change.

[CR40] Seidl R, Baier P, Rammer W, Schopf A, Lexer MJ (2007). Modelling tree mortality by bark beetle infestation in Norway spruce forests. Ecol Model.

[CR41] Seidl R, Fernandes PM, Fonseca TF, Gillet F, Jönsson AM, Merganičová K, Netherer S, Arpaci A, Bontemps JD, Bugmann H, González-Olabarria JR (2011). Modelling natural disturbances in forest ecosystems: a review. Ecol Model.

[CR42] Seidl R, Müller J, Hothorn T, Bässler C, Heurich M, Kautz M (2016). Small beetle, large-scale drivers: how regional and landscape factors affect outbreaks of the European spruce bark beetle. J Appl Ecol.

[CR43] Seidl R, Rammer W, Blennow K (2014). Simulating wind disturbance impacts on forest landscapes: tree-level heterogeneity matters. Environ Model Softw.

[CR44] Seidl R, Rammer W, Scheller RM, Spies TA (2012). An individual-based process model to simulate landscape-scale forest ecosystem dynamics. Ecol Model.

[CR45] Seidl R, Schelhaas M-J, Lindner M, Lexer MJ (2009). Modelling bark beetle disturbances in a large scale forest scenario model to assess climate change impacts and evaluate adaptive management strategies. Reg Environ Change.

[CR46] Seidl R, Schelhaas M-J, Rammer W, Verkerk PJ (2014). Increasing forest disturbances in Europe and their impact on carbon storage. Nat Clim Change.

[CR47] Seidl R, Spies TA, Peterson DL, Stephens SL, Hicke JA (2016). Searching for resilience: addressing the impacts of changing disturbance regimes on forest ecosystem services. J Appl Ecol.

[CR48] Silva Pedro M, Rammer W, Seidl R (2016). A disturbance-induced increase in tree species diversity facilitates forest productivity. Landscape Ecol.

[CR49] Simard M, Lajeunesse P (2015). The interaction between insect outbreaks and debris slides in a glacial valley of the Eastern Canadian Shield. Ecosystems.

[CR50] Simard M, Romme WH, Griffin JM, Turner MG (2011). Do mountain pine beetle outbreaks change the probability of active crown fire in lodgepole pine forests?. Ecol Monogr.

[CR51] Sproull GJ, Adamus M, Bukowski M, Krzyżanowski T, Szewczyk J, Statwick J, Szwagrzyk J (2015). Tree and stand-level patterns and predictors of Norway spruce mortality caused by bark beetle infestation in the Tatra Mountains. For Ecol Manag.

[CR52] Stadelmann G, Bugmann H, Wermelinger B, Bigler C (2014). Spatial interactions between storm damage and subsequent infestations by the European spruce bark beetle. For Ecol Manag.

[CR53] Temperli C, Bugmann H, Elkin C (2013). Cross-scale interactions among bark beetles, climate change, and wind disturbances: a landscape modeling approach. Ecol Monogr.

[CR54] Temperli C, Zell J, Bugmann H, Elkin C (2013). Sensitivity of ecosystem goods and services projections of a forest landscape model to initialization data. Landscape Ecol.

[CR55] Thom D, Rammer W, Dirnböck T, Müller J, Kobler J, Katzensteiner K, Helm N, Seidl R (2016) The impacts of climate change and disturbance on spatio-temporal trajectories of biodiversity in a temperate forest landscape. J Appl Ecol (in press). doi: 10.1111/1365-2664.12644 10.1111/1365-2664.12644 PMC5245768 28111479

[CR56] Turner MG (2010). Disturbance and landscape dynamics in a changing world. Ecology.

[CR57] Turner MG, Baker WL, Peterson CJ, Peet RK (1998). Factors influencing succession: lessons from large, infrequent natural disturbances. Ecosystems.

[CR58] Wermelinger B (2002). Development and distribution of predators and parasitoids during two consecutive years of an *Ips typographus* (Col., Scolytidae) infestation. J Appl Entomol.

[CR59] Wermelinger B (2004). Ecology and management of the spruce bark beetle *Ips typographus*—a review of recent research. For Ecol Manage.

[CR60] Wermelinger B, Seifert M (1999). Temperature-dependent reproduction of the spruce bark beetle *Ips typographus*, and analysis of the potential population growth. Ecol Entomol.

[CR61] Westerling AL, Turner MG, Smithwick EAH, Romme WH, Ryan MG (2011). Continued warming could transform Greater Yellowstone fire regimes by mid-21st century. Proc Natl Acad Sci U S A.

