# Supplementary material for: Climate change amplifies the interactions between wind and bark beetle disturbances in forest landscapes
Source: Landsc Ecol. 2016 May 23;32(7):1485–98. doi: 10.1007/s10980-016-0396-4 (PMC5494037; doi:10.1007/s10980-016-0396-4)
Supplement: Supplementary file 1 — Supplementary material 1 (DOCX 225 kb) [file 10980_2016_396_MOESM1_ESM.docx]

Supplementary Material

**Climate change amplifies the interactions between wind and bark beetle disturbance
in forest landscapes**

Rupert Seidl, Werner Rammer

**Appendix A: Detailed description of the iLand bark beetle module**

***Initiation of an outbreak***

An annual probability for the occurrence of a bark beetle disturbance *P_base_* is derived from the observed disturbance rotation period (DRP) for bark beetles. This can either be specified as a single (i.e., spatially uniform) probability for the entire landscape, but can also be supplied as map that accounts for spatial differences on the simulated landscape. However, even if a spatially uniform outbreak probability is supplied to the model (as done in this study) the realized bark beetle disturbance regime in the simulation will vary strongly for landscapes that feature strong climatic gradients, as a result of the explicit effect of climate on beetle development – see below. To derive *P_base_* for the simulation from DRP, the average size of a bark beetle outbreak area per year (*size*) and the reference size of the calculation (here 100 × 100 m cells) (*area*) need to be accounted for as described in Eq. 1:

$P_{base}=\frac{1}{DRP}\cdot\frac{area}{size}$ Eq. 1

Values for DRP can be derived for instance by means of dendrochronology (e.g., Čada et al. 2013) or from recent disturbance records (e.g., Thom et al. 2013). Temporally, outbreak probabilities vary strongly between years, and recent studies on the multi-scale drivers of bark beetle outbreaks show that regional-scale variation in climate is an important trigger for outbreaks (Seidl et al. 2015). The outbreak probability *P_bb_* is thus derived from *P_base_* by accounting for the climatic conditions of a given year. This is accomplished using a climate-sensitive modifier of bark beetle probability *r_c_*. as described in Eqs 2-4:

${odds}_{base}=\frac{P_{base}}{1-P_{base}}$ Eq. 2

${odds}_{bb}={odds}_{base}\cdot r_{c}$ Eq. 3

$P_{bb}=\frac{{odds}_{bb}}{1+{odds}_{bb}}$ Eq. 4

The climate modifier *r_c_* is related to interannual climate variation at the regional level and is thus uniform over the landscape. It is a measure of relative change, where the baseline (i.e., a value of 1) is related to the climate conditions for which *P_base_* was specified (e.g., the long-term mean climate over the disturbance observation period). It can be determined using climate-sensitive outbreak relationships that are derived either based on detailed modeling analyses by means of meta-modeling (e.g., Seidl et al. 2009), or from empirical analyses of the large-scale climatic drivers of bark beetle outbreaks (e.g., Seidl et al. 2011). For the *P. abies – I. typographus* system currently parameterized in iLand we followed the in-depth analysis of Seidl et al. (2015) and related outbreak probability to regional variation in summer precipitation.

If a wind disturbance event is simulated *P_bb_* is increased by the probability that windfelled and –broken trees are colonized by bark beetles, currently set to 0.30, based on previous empirical analyses (Eriksson et al. 2005; Eriksson et al. 2008). Salvaging windthrown trees can thus strongly dampen the probability of outbreak initiation in the simulation (see also Stadelmann et al. 2013). Bark beetle outbreaks are initiated by drawing a random number and comparing it against *P_bb_*. A prerequisite for initiation at a given location on the landscape is the availability of viable host trees, defined as Norway spruce trees exceeding a certain threshold diameter (here set to 15 cm dbh). The spatial grain of the simulation of outbreak initiation and spread are 10 x 10 m cells (i.e., the approximate area covered by the crown of an old-growth tree (Lexer and Hönninger 2001). For these cells the dominant Norway spruce tree is evaluated in order to determine whether the cell is a potential host cell for the beetle.

***Beetle development***

Bark beetle development is simulated by means of a phenology-based process model (Seidl et al. 2007; Baier et al. 2007). A cumulative sum of maximum air temperature of 140.3 degree days above the beetles’ lower developmental threshold (DTL = 8.3 ◦C; (Wermelinger and Seifert 1998)) accumulated from the April 1^st^ onward is used to predict the onset of spring swarming and first colonization of trees. Beetles only swarm on days with air temperature maxima of >16.5°C. Essential for brood development, however, is bark temperature rather than ambient temperature. To derive bark temperature, a previously established empirical relationship between bark temperature, air temperature, incoming radiation above the canopy, and relative light level (the latter derived here from the local leaf area index simulated in iLand) is employed (Baier et al. 2007). Brood development is estimated using upper and lower temperature thresholds (8.3 and 38.9°C, respectively) and a non-linear function for calculating effective bark temperatures and thermal sums necessary for successful bark beetle development (Wermelinger and Seifert 1998; Wermelinger and Seifert 1999). If the effective bark temperature sum reaches the heat sum of 557 degree days beetle development is completed. If the maximum air temperature exceeds the required lower limit of swarming (16.5°C), and day length is still >14.5 h, beetles disperse (see below) and start sister broods (i.e., new broods established by the initial parental generation of beetles) and/ or a new filial generation. If the accumulated effective bark temperature exceeds beetle 278.5 degree days and the swarming requirements are met a sister brood is initiated. Sister broods develop in analogy to the main filial generations using the same developmental thresholds. Beetle development stops either through bark temperatures below the lower developmental threshold or through day lengths of <14.5 h (Seidl et al. 2007; Baier et al. 2007).

***Beetle dispersal***

Every completed beetle generation disperses from the colonized host if the swarming requirements are met. The tree is killed once the beetles leave the host, and is no longer available as potential host tree for future beetle attacks. All mature Norway spruce trees above the colonization threshold growing on the focal 10 × 10 m cell are assumed to be colonized and killed in the same time step. Spatially explicit beetle dispersal follows a two-step approach:

In a first step, beetle flight follows a symmetrical dispersal kernel (see e.g., Fahse and Heurich 2011; Kautz et al. 2011). The direction of dispersal is randomly chosen and the distance is determined from the probabilistic kernel function. In a second step, the thus determined landing location is further modified by the beetle actively searching for potential hosts in the vicinity. The perceptual range of the beetles for this search was previously estimated to be in the range of 15 m (Fahse and Heurich 2011; Kautz et al. 2014). We here used the Moore neighborhood around the initial landing cell of the beetle to constrain the beetles search for potential hosts. If no potential host is present in this 30 × 30 m area the beetle cohort is assumed to die (dispersal mortality). If both living and freshly windthrown host trees are available, the beetles preferentially colonize the latter. If selected by managers, salvage harvesting of windthrown trees is assumed to occur before the brood of the colonizing beetles leaves the tree, and thus reduces the local bark beetle pressure. Trap trees, frequently used to monitor beetle development and reduce local beetle pressure by managers, are treated as freshly windthrown trees, but are assumed to be removed from the site before the next beetle generation can emerge in the simulation.

The aggregation behavior of beetles via their chemical communication is not simulated explicitly in iLand. Rather than simulating the dispersal of pioneer beetles and the subsequent attraction of followers via pheromones, which eventually exhausts tree defenses and leads to a successful attack (Wermelinger 2004; Kausrud et al. 2012), we here aggregate this dynamics by simulating the dispersal of beetle cohorts, with a cohort defined as the minimum number of beetles that are jointly able to kill a tree (estimated to be 30 beetles in the case of *I. typographus*, (Kautz et al. 2014)). Every cell for which a successful brood is simulated disperses multiple beetle cohorts, with the number determined by the reproductive success of the beetle. For *I. typographus*, for instance, it can be assumed that 45 larvae are hatched per female colonizing beetle (Fahse and Heurich 2011), representing the upper limit of the reproductive rate. However, if also mortality and density-dependent competition for resources within a brood are considered, the reproductive rate of beetles is likely considerably lower than that (estimated to range between 4 and 24 by Wermelinger and Seifert (1999), and set to 20 for this study). The reproductive success is a user-defined parameter in the model that can be parameterized based on empirical data and specified separately for main generations and sister broods.

***Host colonization***

A beetle cohort attacking a tree has to overcome the trees defense system, including stored and induced resin production as well as induced wound reactions (Baier 1996; Wermelinger 2004). These defense mechanisms are closely related to the availability of non-structural carbohydrates in the tree, and thus bark beetle susceptibility is frequently found to be related to drought stress and low tree vigor (Christiansen et al. 1987; Netherer et al. 2015). iLand dynamically simulates tree stress based on the carbon balance of a tree (Seidl et al. 2012), and the thus derived stress index (where 0 means no stress and a full carbohydrate reserve, while 1 indicates high stress and a fully depleted carbohydrate reserves pool) is used as an indicator for tree defense in the simulation of bark beetle host colonization. Kautz et al. (2014) suggested that a healthy, vigorous tree (i.e., a host tree at its maximum defense capacity) requires 6.67 times more attacking beetles to be successfully colonized compared to a stressed tree. Consequently, we here formulated the probability of successful colonization (*P_colonize_*) by a single beetle cohort (representing the aggregation of 30 beetles) to (Eq. 5)

$P_{colonize}=0.85\cdot SI+0.15$ Eq. 5

with SI is the C-balance derived stress index [0,1]. Windthrown trees are assumed to be essentially defenseless, and area assigned a *P_colonize_* of 1.0. A uniform random number is compared against *P_colonize_* to determine whether a tree is successfully colonized by an attacking cohort of beetles. Please note that during any given dispersal wave multiple beetle cohorts can attack a tree.

***Overwintering of the beetle***

Only the last beetle generation developing in a year is assumed to overwinter. Of that generation, all immature beetles are assumed to experience 100% winter mortality (Faccoli 2002; Jönsson et al. 2012). For mature beetles, simulated winter mortality consists of two components: First, a background winter mortality rate (*M_bg_*) is assumed, i.e., a fixed proportion of adult beetles is killed every winter (here set to *M_bg_*= 0.40, following Jönsson et al. (2012)). Second, additional mortality is caused by exposure to strong winter frost. Specifically, days with minimum temperatures below -15 °C are assumed as frost days (*fd*), and beetle mortality (*M_w_*) increases with days of exposure (Eq. 6):

$M_{w}=1-e^{a_{1}\cdot fd}$ Eq. 6

The empirical parameter *a_1_* was estimated to be -0.1005 by Koštál et al. (2011) under wet conditions, with more than 95% of the beetles dying from 30 days of frost exposure.

***Collapse of outbreaks***

How and why bark beetle outbreaks stop is not yet fully understood. However, observations for the *P. abies – I. typographus* system show that regional eruptions of beetles die down regularly after six years on average (Kautz et al. 2011; Tomiczek et al. 2012; Lausch et al. 2013; Seidl et al. 2015). Antagonists, such as predatory beetles (*Cleridae*) and flies (*Dolichopodidae*), as well as parasitic wasps (*Pteromalidae, Braconidae*) and birds, play an important role in mitigating and containing bark beetle outbreaks (Wermelinger 2002; Wermelinger 2004). A further aspect in the collapse of outbreaks is likely to be a decreased fitness of individual beetles due to intraspecific competition and density-dependent feedbacks (Marini et al. 2013; Kautz et al. 2014). However, modeling population dynamics of the diverse community of antagonists as well as the fitness level of individual beetles was beyond the scope of the iLand bark beetle disturbance module. Consequently, we assumed a fixed negative feedback in later stages of bark beetle outbreaks, parameterized to mimic the observed pattern of periodic collapses of bark beetle populations. Specifically, we defined the effect of negative feedbacks (as represented by an additional beetle mortality component *M_nf_*) to depend on the time elapsed since the initiation of an outbreak spot (*t_ob_*), following Eq. 7,

$M_{nf}=min(\max\left( 2\cdot\frac{t_{ob}}{t_{max}}-1,0 \right),0)$ Eq. 7

with *t_max_* an empirical parameter randomly selected for each year between 5 and 6 to mimic the observed periodicity of outbreaks in the *P. abies – I. typographus* system.

**Appendix B: Parameters and sensitivity analyses**

**Parameters of the bark beetle module**

Table B1 lists the parameters of the bark beetle module, along with the values that were used in this study. In addition, the table describes the rationale for choosing these parameter values and gives the respective references.

Table B1. Parameters for the bark beetle module used in this study as well as the literature sources for the values.

| **Parameter** | | **Value** | **Description** |
| --- | --- | --- | --- |
| ***Initiation of an outbreak*** | | |  |
| *P_base_* | | 0.000685 | Annual outbreak probability per hectare.  Estimated from a disturbance rotation period of 365 years and a mean disturbance size of 4 ha (Thom et al. 2013; Thom et al. 2016). |
| *r_c_* | | ${P_{summer,rel}}^{-0.9609}$ | Climate sensitive modifier of outbreak probability.  Based on a reanalysis of data given in (Seidl et al. 2015). *P_summer,rel_* is the relative summer precipitation (JJA) of the previous year relative to the long-term average. |
| *P_windthrown_* | | 0.3 | Probability of bark beetles colonizing windthrown trees. Based on empirical data from Eriksson et al. (2005) and Eriksson et al. (2008). |
| ***Beetle dispersal*** | | |  |
| *K_spread_* | | $e^{-\frac{\left( \frac{x}{4.5} \right)^{2}}{40.5\cdot4}}$ | Bark beetle dispersal kernel, calculating the probability that beetles spread to *x* m from the center of the originating cell. The thus defined kernel is scaled to a sum of 1. The kernel function is taken from Fahse and Heurich (2011) (see also Kautz et al. (2011) for alternative kernel functions, which, however, include the compounding spread of multiple generations per year). |
| *N_cohorts, main_* | | 20 | Reproduction rate of the beetle, i.e. the number of beetle cohorts spreading from infested pixels for each main beetle generation. Empirical evidence points to values between 4 and 24 (Wermelinger and Seifert 1999), with a theoretical upper limit of 45 (Fahse and Heurich 2011). |
| *N_cohorts, sisterbroods_* | | 30 | Reproduction rate of beetles if - in addition to the filial generation - a full sister brood develops.  It is assumed that the reproductive rate in sister broods is 50% lower than that of a main generation. |
| ***Host colonization*** | | |  |
| *DBH_min_* | | 15 | Threshold diameter at breast height for colonization (cm). Cells occupied by Norway spruce tree above the threshold are considered as potential host cells. |
| *P_colonize_* | | $0.85\cdot SI+0.15$ | Probability of successful colonization by one beetle cohort as a function of SI, the C-balance derived stress index (Seidl et al. 2012). Following Kautz et al. (2014) it is assumed that healthy trees require 6.7 times more beetles for successful colonization compared to stressed trees. |
| ***Overwintering of the beetle*** | | |  |
| *M_bg_* | | 0.4 | Background winter mortality rate (Jönsson et al. 2012). |
| *M_w_* | | $1-e^{-0.1005\cdot fd}$ | Additional probability of beetle mortality due to days with minimum temperature below -15°C, following Koštál et al. (2011). |
| ***Collapse of outbreaks*** | | |  |
| *M_nf_* | $min(\max\left( 2\cdot\frac{t_{ob}}{t_{max}}-1,0 \right),0)$ | | Additional beetle mortality due to antagonists and decreased fitness of beetles in later stages of an outbreak. *t_ob_* is the time elapsed (in years) since the initiation of an outbreak spot. *t_max_* is the maximum duration of an outbreak and is selected randomly each year from an user-specified interval, here set to [5,6]. Sources: Kautz et al. (2011); Tomiczek et al. (2012); Seidl et al. (2015). |

**Sensitivity analysis to parameter changes**

We conducted a sensitivity analysis in order to gain insights into the responses of the newly developed bark beetle module to changes in key parameters. We followed a “one-at-a-time” approach, i.e. we changed the value of one parameter at a time while all other parameters remained at their default values (local sensitivity analysis). In order to investigate the model behavior with regard to the initiation of outbreaks and the spread of bark beetles, we chose the infestation probability for windthrown trees (*P_windthrown_*), the number of beetle cohorts (*N_cohorts_*), the maximum outbreak duration (*t_max_*), the minimum diameter at breast height for potential host trees (*DBH_min_*), and the bark beetle spread distance (as expressed by the spread kernel function *K_spread_*) as parameters for the analysis (Table B2). Whenever possible, parameters were set to +20% and -20% of their base value (see Table B1). As output indicator we selected the total simulated area disturbed (including both bark beetle and wind disturbance). The simulation setup was identical as the setup described in the main text, with simulations running from 2007-2014 under baseline climate conditions.

Table B2. Description of default and alternative values for the parameters analyzed in the sensitivity analysis.

| Parameter | Change | standard value | Modified value (low) | Modified value (high) |
| --- | --- | --- | --- | --- |
| Reproduction rate *N_cohorts,base_/ N_cohorts,sisterbroods_* | ±20% | 20/30 | 16/24 | 24/36 |
| Infestation probability for windthrown trees *P_windthrown_* | ±20% | 0.3 | 0.24 | 0.36 |
| Maximum duration of bark beetle outbreaks *t_max_* (range) | ±1 year | 5/6 | 4/5 | 6/7 |
| Minimum tree diameter (*DBH_min_*) | ±20% | 15 | 12 | 18 |
| Beetle spread distance^1^ in meter | ±20% | 132 | 105.6 | 158.4 |

^1)^ the parameter *d* in the kernel function ${K_{spread}=e}^{-\frac{\left( \frac{x}{4.5} \right)^{2}}{d\cdot4}}$ (see also Table B1) was modified in such a way that the radius within which 99.9% of all beetles landed was changed by +-20%. The used values for *d* values were 40.5, 27, and 59, respectively.

Table B3 shows the results of the sensitivity analysis. With the exception of *DBH_min_*, the model simulated increased damage with increasing parameter values. The model showed the highest sensitivity to a ±20% change in the reproduction rate of the beetle (+85%, -42%). We also found a high sensitivity to changes in the maximum duration of the bark beetle outbreak (+49%, - 22%), and slightly lower responses to *d_spread_* (+22%, -18%), *P_windthrown_* (+31%, -3%), and *DBH_min_* (-6.3%, +13.4%).

Table B3. Results of changing key parameters of the bark beetle module in a one-at-a-time sensitivity analysis (see also Table B2). Shown is the total disturbed area (ha) between 2007 and 2014. The base value of the model running with default parameters was 531.7 ha.

| Paramter | Lower value | Higher value |
| --- | --- | --- |
| *N_cohorts_* | 306.6 | 987.5 |
| *P_windthrown_* | 513.2 | 696.4 |
| *t_max_* | 414.2 | 791.4 |
| *DBH_min_* | 602.9 | 498.4 |
| *d_spread_* | 437.0 | 649.3 |

**Appendix C: Sensitivity to inter-annual climate variability**

In addition to the one-at-a-time sensitivity analysis (Appendix B) we investigated the sensitivity of the bark beetle module to inter-annual variations in climatic drivers. Climate influences the simulated bark beetle damage directly through three pathways: First, the number of beetle generations developed per year is simulated based on phenology, and thus mainly driven by the temperature regime from April to September. Second, the probability of new outbreaks (in the absence of wind disturbances) increases with dry summers of the previous year. And third, drought-induced stress of potential host trees reduces their ability of trees to defend themselves against attacking bark beetles. In this sensitivity analysis we focus on the direct effect of climate on the simulated number of bark beetle generations per year. Figure C1 shows the number of generations that was simulated for the Kalkalpen Nationalpark (KANP) landscape for the years 2007-2014. For 2007, the year of the storm Kyrill, 62.4% of the KANP landscape were able to sustain more than two generations (i.e., either two filial and sisterbrood generations, or three filial generations). In contrast, in 2010, the area supporting the development of more than two generations dropped to 10.8% (Figure C2). It is interesting to note, that overall the mean temperature of the analyzed period 2007-2014 was already 1.3°C higher than that of the period 1970-2000.

To further elucidate the effect of climate variability, we ran a number of simulations where we fixed the climate to a specific year and analyzed the resulting total area disturbed in the simulation including wind and bark beetle disturbance. For this test we selected the years with the most (2007) and the median (2013) number of bark beetle generations within the 2007-2014 period. Furthermore we included a typical year from the period 1970-2000 (the year with the median annual temperature, i.e. 1977) as additional benchmark. Simulations with the median year (2013) result in an increase of the total disturbed area of 1.8% compared to a simulation with annually varying climate (Table C1). If temperatures were persistently as high as 2007 throughout the eight year simulation period, the total disturbance damage more than doubled to 1229.1 ha. In contrast, the median year of the period 1970-2000 resulted in a reduction of the disturbed area by 26% compared to the default run considering full interannual climate variability. These results highlight not only the climate sensitivity of the bark beetle module, but also illustrate the effect of temperature on the simulated wind disturbance: Despite assuming identical wind speeds, the disturbed area due to storms was considerably lower for runs with both constant climate at the levels of 2013 and 1977, which was related to a larger area of the landscape experiencing frozen soils at the respective days of the wind events. Trees on frozen soils have better anchorage (Usbeck et al. 2010), and are assumed to be only susceptible to stem breakage in the model (Seidl et al. 2014).

Figure C1. Maps of the number of simulated bark beetle generations at KANP for the years 2007 to 2014. The color-coded values indicate the number of filial generations. 0.5 is added if a sisterbrood can develop in addition to the last filial beetle generation (e.g., 1.5=one generation and one sisterbrood).

Figure C2. Distribution of the simulated number of bark beetle generations in percent of the total area for the warmest (2007) and coolest (2010) year of the period 2007-2014. The categories “1+” and “2+” denote areas where a sisterbrood can develop in addition to the respective filial generations.

Table C1. Simulated area disturbed during the eight year study period disregarding inter-annual climate variability and imposing the climate of a single year for the entire study period. The reference value for the total disturbed area using the default climate is 531.1 ha (wind: 180.3 ha, bark beetles 351.4 ha).

| Year | Total area disturbed (ha) | | |
| --- | --- | --- | --- |
|  | Wind | Bark beetle | Total |
| 2013 (median 2007-2014) | 131.4 | 409.7 | 541.1 |
| 2007 (warmest year) | 173.3 | 1055.8 | 1229.1 |
| 1977 (median 1970-2000) | 83.2 | 309.2 | 392.4 |

**References Appendices A-C**

Baier P (1996) Defence reactions of Norway spruce (Picea abies Karst.) to controlled attacks of Ips typographus (L.) (Col., Scolytidae) in relation to tree parameters. J Appl Entomol 120:587–593. doi: 10.1111/j.1439-0418.1996.tb01656.x

Baier P, Pennerstorfer J, Schopf a (2007) PHENIPS—A comprehensive phenology model of Ips typographus (L.) (Col., Scolytinae) as a tool for hazard rating of bark beetle infestation. For Ecol Manage 249:171–186. doi: 10.1016/j.foreco.2007.05.020

Čada V, Svoboda M, Janda P (2013) Dendrochronological reconstruction of the disturbance history and past development of the mountain Norway spruce in the Bohemian Forest, central Europe. For Ecol Manage 295:59–68. doi: 10.1016/j.foreco.2012.12.037

Christiansen E, Waring RH, Berryman AA (1987) Resistance of conifers to bark beetle attack: Searching for general relationships. For Ecol Manage 22:89–106. doi: 10.1016/0378-1127(87)90098-3

Eriksson M, Neuvonen S, Roininen H (2008) Ips typographus (L.) attack on patches of felled trees: “Wind-felled” vs. cut trees and the risk of subsequent mortality. For Ecol Manage 255:1336–1341. doi: 10.1016/j.foreco.2007.10.043

Eriksson M, Pouttu A, Roininen H (2005) The influence of windthrow area and timber characteristics on colonization of wind-felled spruces by Ips typographus ( L .). 216:105–116. doi: 10.1016/j.foreco.2005.05.044

Faccoli M (2002) Winter mortality in sub-corticolous populations of Ips typographus (Coleoptera, Scolytidae) and its parasitoids in the south-eastern Alps. Anzeiger fur Schädlingskd 75:62–68. doi: 10.1034/j.1399-5448.2002.02017.x

Fahse L, Heurich M (2011) Simulation and analysis of outbreaks of bark beetle infestations and their management at the stand level. Ecol Modell 222:1833–1846. doi: 10.1016/j.ecolmodel.2011.03.014

Jönsson AM, Schroeder LM, Lagergren F, et al (2012) Guess the impact of Ips typographus—An ecosystem modelling approach for simulating spruce bark beetle outbreaks. Agric For Meteorol 166-167:188–200. doi: 10.1016/j.agrformet.2012.07.012

Kausrud K, Økland B, Skarpaas O, et al (2012) Population dynamics in changing environments: the case of an eruptive forest pest species. Biol Rev 87:34–51. doi: 10.1111/j.1469-185X.2011.00183.x

Kautz M, Dworschak K, Gruppe A, Schopf R (2011) Quantifying spatio-temporal dispersion of bark beetle infestations in epidemic and non-epidemic conditions. For Ecol Manage 262:598–608. doi: 10.1016/j.foreco.2011.04.023

Kautz M, Schopf R, Imron MA (2014) Individual traits as drivers of spatial dispersal and infestation patterns in a host–bark beetle system. Ecol Modell 273:264–276. doi: 10.1016/j.ecolmodel.2013.11.022

Koštál V, Doležal P, Rozsypal J, et al (2011) Physiological and biochemical analysis of overwintering and cold tolerance in two Central European populations of the spruce bark beetle, Ips typographus. J Insect Physiol 57:1136–1146. doi: 10.1016/j.jinsphys.2011.03.011

Lausch A, Heurich M, Fahse L (2013) Spatio-temporal infestation patterns of Ips typographus (L.) in the Bavarian Forest National Park, Germany. Ecol Indic 31:73–81. doi: 10.1016/j.ecolind.2012.07.026

Lexer MJ, Hönninger K (2001) A modified 3D-patch model for spatially explicit simulation of vegetation composition in heterogeneous landscapes. For Ecol Manage 144:43–65.

Marini L, Lindelöw Å, Jönsson AM, et al (2013) Population dynamics of the spruce bark beetle: a long-term study. Oikos 122:1768–1776. doi: 10.1111/j.1600-0706.2013.00431.x

Netherer S, Matthews B, Katzensteiner K, et al (2015) Do water-limiting conditions predispose Norway spruce to bark beetle attack? New Phytol 205:1128–1141. doi: 10.1111/nph.13166

Seidl R, Baier P, Rammer W, et al (2007) Modelling tree mortality by bark beetle infestation in Norway spruce forests. Ecol Modell 206:383–399. doi: 10.1016/j.ecolmodel.2007.04.002

Seidl R, Müller J, Hothorn T, et al (2015) Small beetle, large-scale drivers: how regional and landscape factors affect outbreaks of the European spruce bark beetle. J Appl Ecol in press:doi: 10.1111/1365–2664.12540. doi: 10.1111/1365-2664.12540

Seidl R, Rammer W, Blennow K (2014) Simulating wind disturbance impacts on forest landscapes: Tree-level heterogeneity matters. Environ Model Softw 51:1–11. doi: 10.1016/j.envsoft.2013.09.018

Seidl R, Rammer W, Scheller RM, Spies TA (2012) An individual-based process model to simulate landscape-scale forest ecosystem dynamics. Ecol Modell 231:87–100. doi: 10.1016/j.ecolmodel.2012.02.015

Seidl R, Schelhaas M-J, Lexer MJ (2011) Unraveling the drivers of intensifying forest disturbance regimes in Europe. Glob Chang Biol 17:2842–2852. doi: 10.1111/j.1365-2486.2011.02452.x

Seidl R, Schelhaas M-J, Lindner M, Lexer MJ (2009) Modelling bark beetle disturbances in a large scale forest scenario model to assess climate change impacts and evaluate adaptive management strategies. Reg Environ Chang 9:101–119. doi: 10.1007/s10113-008-0068-2

Stadelmann G, Bugmann H, Meier F, et al (2013) Effects of salvage logging and sanitation felling on bark beetle (Ips typographus L.) infestations. For Ecol Manage 305:273–281. doi: 10.1016/j.foreco.2013.06.003

Thom D, Rammer W, Dirnböck T, et al (2016) The impacts of climate change and disturbance on spatio-temporal trajectories of biodiversity in a temperate forest landscape. J Appl Ecol in revision.

Thom D, Seidl R, Steyrer G, et al (2013) Slow and fast drivers of the natural disturbance regime in Central European forest ecosystems. For Ecol Manage 307:293–302.

Tomiczek C, Cech TL, Fürst A, et al (2012) Waldschutzsituation 2011 in Österreich. Forstschutz Aktuell 56:3–10.

Usbeck T, Wohlgemuth T, Dobbertin M, et al (2010) Increasing storm damage to forests in Switzerland from 1858 to 2007. Agric For Meteorol 150:47–55. doi: 10.1016/j.agrformet.2009.08.010

Wermelinger B (2002) Development and distribution of predators and parasitoids during two consecutive years of an Ips typographus (Col., Scolytidae) infestation. J Appl Entomol 126:521–527. doi: 10.1046/j.1439-0418.2002.00707.x

Wermelinger B (2004) Ecology and management of the spruce bark beetle Ips typographus—a review of recent research. For Ecol Manage 202:67–82. doi: 10.1016/j.foreco.2004.07.018

Wermelinger B, Seifert M (1998) Analysis of temperature dependent development of the spruce bark beetle Ips typographus (L.) (Col. Scol.). J Appl Entomol 122:185–191.

Wermelinger B, Seifert M (1999) Temperature-dependent reproduction of the spruce bark beetle Ips typographus, and analysis of the potential population growth. Ecol Entomol 24:103–110. doi: 10.1046/j.1365-2311.1999.00175.x
